# The Mechanism and Design Principles of Serious Games in Enhancing Adolescents’ Internet Adaptability

**DOI:** 10.2196/82505

**Published:** 2026-03-09

**Authors:** Shijie Gao, Min Jia, Weijun Wang, Jianping Hu, Shihao Ma

**Affiliations:** 1 School of Psychology, Central China Normal University, No.152 Luoyu Road, Hongshan District, Wuhan, Hubei, 430079, China, 86 18560177867; 2 Key Laboratory of Adolescent Cyberpsychology and Behavior, Ministry of Education, Wuhan, Hubei, China

**Keywords:** game design mechanism, adolescents, internet adaptability, game based learning, game design principles

## Abstract

Adolescents’ internet adaptability (IA) is crucial for their online behavior and mental health. Serious games (SGs), as an emerging educational tool, hold promise for enhancing this adaptability through engaging, goal-oriented learning. Yet, direct evidence in this area remains limited. This viewpoint aims to clarify the mechanisms through which SGs enhance adolescents’ IA and to derive corresponding design principles that can inform educational practice and game development. Drawing on insights from both Chinese and international studies, this study adopts a cross-contextual perspective to explore how SGs can foster IA in varied educational environments. Beyond synthesizing existing findings, this viewpoint provides an integrated account of why IA is essential in contemporary digital life and how SGs can support its development. It proposes a 3-stage framework, illustrating how contextualized design, real-time feedback, and dynamic tasks promote experiential learning, self-regulation, and the transfer of online skills. Based on this framework, the study further articulates 6 core design principles: clear goal definition, interaction diversity, contextual authenticity, immediate, scaffolding and explanatory feedback, a dynamically adaptive learning environment, and safety-by-design for digital well-being. These principles translate the core characteristics and mechanisms of SGs into actionable guidance for developing effective IA interventions. By synthesizing theoretical insights with practical considerations, this viewpoint highlights how SGs can serve as accessible and scalable tools to support adolescents in navigating increasingly complex digital environments. Together, these insights provide practical implications for educators, curriculum designers, and digital game developers seeking to foster adolescents’ safe, responsible, and adaptive engagement in online environments.

## Introduction

**Figure 1.**
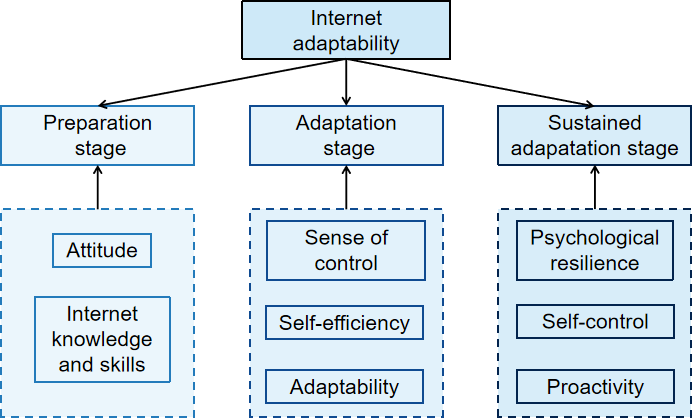
The conceptualization of internet adaptability.

Internet adaptability (IA) refers to the individual capacities that develop through interactions with the online environment [1]. It is a core component of digital engagement and develops across three stages: (1) the preparation stage, in which adolescents acquire relevant knowledge and psychological readiness; (2) the adaptation stage, involving their evaluation of the digital environment and behavioral adjustment; and (3) the sustainability stage, reflecting their ability to cope with technological change and digital stressors [1,2]. The following conceptualization illustrates these 3 stages and the psychological factors that characterize each [1] (Figure 1).

To clarify its conceptual boundaries, IA must be distinguished from related constructs. Digital literacy or competence emphasizes relatively stable technical skills such as information processing and tool use [3-5]. Digital resilience focuses on recovering from adverse online experiences and is primarily reactive [6]. Digital well-being concerns maintaining a healthy and balanced relationship with technology [7]. In contrast, IA represents a dynamic psychological adjustment process, grounded in self-efficacy, sense of control, resilience, and proactive regulation, that supports sustained functional engagement across evolving digital contexts [1,8]. A comparative overview of these constructs is presented in Table 1.

**Table 1.** Internet adaptability and related concepts.

Concepts	Definitions	Static or dynamic	Main dimensions	Relationship with IA^a^
Internet adaptability [1]	Individual capacities that develop through interactions with the online environment.	Dynamic and process-oriented	Attitude, online knowledge and skills, sense of control, self-efficacy, adaptability, self-control, psychological resilience, and proactivity.	IA integrates elements from digital competence, resilience, and well-being but distinguishes itself by focusing on long-term adaptation, proactive regulation, and psychological behavioral flexibility in a changing digital environment.
Digital literacy or competence [3,4]	Competence in using digital technologies to access, evaluate, create, and communicate information; ability to use ICT^b^ tools appropriately.	More on stable skill set or capacity (relatively static)	Information and data literacy, communication or collaboration, content creation, safety or cybersecurity, problem solving, critical thinking, and ethical or reflective use.	IA differs by emphasizing ongoing adaptation and psychological–behavioral regulation, not just ability to use tools.
Digital resilience [6]	The capacity to cope with, recover from, and learn after adverse or risky online events or digital pressures; ability to bounce back, maintain well-being, and continue functioning after negative experiences.	More reactive or recovery-oriented (although may include learning after adversity)	Coping strategies, recovery, learning from negative online experiences, psychological well-being, and safe behavior online.	IA encompasses but extends beyond DR^c^: while DR addresses response to adversity, IA emphasizes proactive, continuous adaptation and regulation across changing digital contexts.
Digital well-being [7]	Individual’s ability to establish a healthy, balanced, and purposeful relationship with digital technologies, thereby supporting their overall quality of life and personal development.	Ongoing state-oriented or stability-oriented	Digital habits, healthy use, ethical awareness, balanced usage, mental health, life–digital balance, and critical media use.	IA emphasizes adaptive capabilities (cognitive, behavioral, and psychological) to manage digital environments; well-being is an important outcome, but IA focuses more on adaptive functioning, not only well-being.

a IA: internet adaptability.

b ICT: information and communication technology.

c DR: digital resilience.

A key question is whether IA applies across different cultural internet environments. From a social adaptation perspective, development reflects the dynamic balance between personal characteristics and environmental demands [9]. Similarly, IA describes the psychological process through which individuals regulate and adjust behavior in response to changing online conditions. Although digital ecosystems differ across cultures, the core adaptive task remains constant: maintaining functional and psychological balance in a dynamic digital environment [1,10]. Thus, IA captures a universal adaptive mechanism, even as specific challenges and adaptive behaviors vary across societies.

IA plays an important role in adolescents’ psychological well-being and digital engagement [1,11]. However, current approaches to fostering IA, including classroom instruction, family education, campus initiatives, and internet use regulations, often lack contextual relevance, interactivity, and flexibility, limiting their effectiveness in addressing adolescents’ evolving online challenges [12].

Serious games (SGs) have emerged as a promising alternative. Originating from board games, they integrate education with entertainment to provide personalized and interactive learning experiences [13]. With advances in digital technology and learning theory, SGs have evolved into formats such as video games, simulations, and virtual reality environments. This study focuses on educational video games, defined as games designed for purposes beyond entertainment [14,15]. In this context, SGs refer to educational video games that integrate gaming elements to transmit knowledge, train skills, and promote emotional or behavioral development in a context-rich and engaging way [16,17]. Well-designed SGs typically exhibit several core characteristics: clear educational objectives [18], disciplinary rigor [19], contextual authenticity [20], and interactivity with immediate formative feedback [21]. When integrated effectively, these features support engagement while promoting meaningful learning and behavioral development.

These immersive and interactive affordances make SGs particularly suitable for fostering adolescents’ IA. By embedding learning goals within gameplay and simulating realistic online scenarios, SGs enable adolescents to develop cognitive, emotional, and behavioral regulation in controlled virtual environments, supporting safe, responsible, and flexible digital engagement. Empirical studies support these benefits. For example, SGs targeting cyberbullying prevention enhance empathy and emotional regulation [22], and the cybersecurity game *Riskio* strengthens awareness and perceived control under uncertain conditions [23-25]. Extending beyond isolated dimensions, *InterWeb Action*, a SG grounded in situated learning theory, significantly improved overall IA and multiple behavioral indicators [26].

Despite these emerging findings, research that directly and systematically examines how SGs cultivate IA remains limited. Therefore, rather than conducting a conventional literature review, this viewpoint synthesizes existing evidence to articulate the mechanisms through which SGs may enhance IA and to propose practical design principles for effective intervention development.

**Figure 2.**
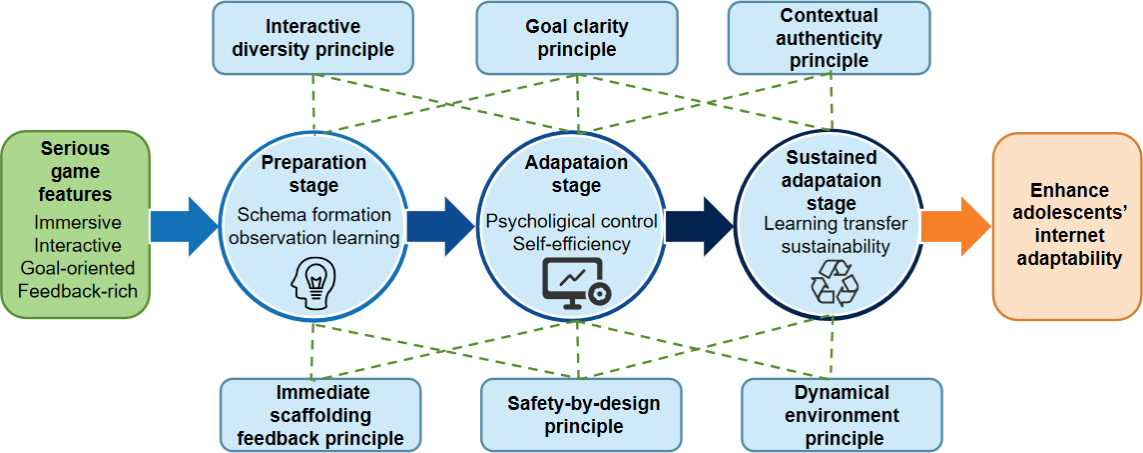
Conceptual framework of the study.

Building on this aim, the viewpoint advances a theoretical framework that examines the psychological processes involved in IA across 3 stages and derives 6 core design principles that translate theory into actionable guidance for educators, curriculum designers, and game developers seeking to promote safe, responsible, and adaptive online behavior (Figure 2).

## The Unique Potential of SGs for Adolescents’ IA

### The Universality of SGs in Educational Practice

Although research directly examining SGs for enhancing adolescents’ IA remains limited, their educational effectiveness has been widely demonstrated across diverse learning contexts [27,28].

Meta-analytic evidence indicates that SGs enhance cognitive performance while simultaneously promoting positive emotions and well-being during learning. Empirical studies further show that SGs improve academic achievement and learning motivation across disciplines such as science, language learning, and business education [29,30].

Beyond cognitive outcomes, SGs also support the development of soft skills. For example, the SG *FLIGBY* has been shown to foster leadership, conflict resolution, and critical thinking, highlighting the role of SGs in holistic skill development [31]. Compared with traditional instructional approaches, SGs offer greater flexibility and learner autonomy, aligning well with contemporary educational needs and contributing to more positive learning attitudes [28,32]. Collectively, these findings establish SGs as an effective and versatile educational tool, providing a strong foundation for their application in promoting adolescents’ IA.

### The Relevance of SG Features to Adolescents’ IA

As adolescents face increasingly complex digital environments, SGs offer particular advantages due to their educational orientation, immersive contexts, and interactive design. Although direct empirical evidence remains limited, existing studies suggest several pathways through which SGs may support IA.

#### Purpose Fit: Precise Alignment of Educational Goals and Needs

SGs enable a close alignment between educational objectives and learners’ needs. By embedding learning goals within game tasks and challenges, SGs effectively convey internet-related knowledge while fostering cognitive skills such as critical thinking and problem solving [27,28]. This goal-oriented design makes SGs well suited for strengthening adolescents’ online knowledge and competencies.

#### Environmental Adaptation: A Bridge Between Virtual Scenarios and Real-World Issues

SGs provide simulated digital environments that bridge virtual experiences and real-world online challenges. Through scenario-based gameplay, adolescents can practice identifying cybersecurity risks, managing information overload, and protecting personal privacy in a safe and controlled setting. Such contextualized experiences enhance immersion and facilitate the transfer of learned skills to real online environments [33].

#### Formal Innovation: Dual Enhancement of Engagement and Learning Outcomes

The formal design of SGs enhances both engagement and learning outcomes. Rich audiovisual elements, narrative structures, and interactive feedback mechanisms increase adolescents’ motivation and sustained participation [19]. The combination of contextual authenticity and interactivity further strengthens experiential learning and supports behavioral adaptation [26].

In summary, SGs integrate knowledge acquisition, emotional engagement, and behavioral practice within interactive digital contexts, closely aligning with the multidimensional nature of IA. Despite these theoretical and practical advantages, empirical research explicitly examining how SGs foster adolescents’ IA remains scarce [26]. To address this gap, the following section examines the psychological mechanisms through which SGs support IA across the stages of preparation, adaptation, and sustained adaptation, thereby laying the foundation for the proposed design principles.

## A 3-Phase Dynamic Mechanism Model: How SGs Facilitate Adolescents’ IA

Building on the alignment between SGs and IA, we propose a 3-phase dynamic mechanism explaining how SGs support adolescents’ adaptive development online. The model views IA as a trait shaped through ongoing interaction with digital environments, reflecting a reciprocal individual–context relationship [1]. It includes 3 stages: preparation, adaptation, and sustained adaptation, through which SGs promote continuous improvement in adolescents’ ability to navigate the online world.

### The Role of SGs in the Preparation Stage

In the preparation stage of IA, SGs primarily support adolescents in building foundational internet knowledge and skills while cultivating constructive attitudes toward digital environments. These early cognitive and affective structures form the basis for subsequent adaptive behaviors.

Adolescents need to develop a positive internet attitude, including cognitive, emotional, and behavioral tendencies, which influence responsible online behavior [34,35]. Simultaneously, internet knowledge and skills, such as information search, online communication, and cybersecurity practices, are essential for online activities [1]. These constructs interact: a positive attitude motivates learning, while improved skills strengthen engagement and perception of the internet [36].

SGs are well positioned to facilitate this preparatory learning process. Their immersive, interactive, and narrative-rich environments enable learners to construct initial cognitive frameworks about the online world [32]. By integrating technology, pedagogy, and interactive mechanisms [37], these games provide contextualized learning experiences that make abstract concepts concrete and engaging [38]. From the perspective of schema theory, such contextualized learning environments are particularly effective for supporting the formation and refinement of cognitive schema [39]. Schemas function as early cognitive structures that organize new information, shape expectations, and influence attitudes. Some studies emphasize that learners encode and organize information more efficiently when it is embedded in meaningful, situational contexts that can be connected to prior knowledge [40-42]. Contextualized learning allows adolescents to connect new information to prior knowledge, promoting deeper understanding and stable orientations toward online environments.

Observational learning further reinforces early learning. According to Bandura’s social cognitive theory, individuals acquire knowledge, attitudes, and behavioral strategies by attending to models and evaluating the consequences of their actions [43]. This process is supported by four subprocesses (attention, retention, reproduction, and motivation), which together determine how modeled behaviors are internalized [43]. Within SGs, these subprocesses are activated through designed modeling features. For example, non-player characters, scripted demonstrations, or peer-like avatars provide examples of digital norms, information-seeking strategies, and responses to common online dilemmas [21]. Empirical studies indicate that observing modeled responses in interactive digital environments can improve learners’ ability to identify risks [23], foster more prosocial online attitudes [44,45], and support the development of normative expectations for digital interactions [46].

During the preparation stage, observational learning shapes early cognitive and affective orientations. Modeled experiences provide structured exposure to desirable online behaviors, allowing adolescents to internalize norms before independently navigating online environments.

Through the integrated mechanisms of schema formation and observational learning, SGs provide a safe, immersive learning environment. This enables adolescents to effectively construct internet knowledge and skills, cultivate a positive attitude, and actively explore the online environment, thereby laying a solid cognitive and affective foundation for the development of robust IA.

### The Role of SGs in the Adaptation Process Stage

Beyond mastering internet skills, adolescents need a sense of control over the online environment. During adaptation, SGs help develop this psychological control and enhance self-efficacy, fostering confidence in handling online challenges.

Sense of control refers to one’s belief in influencing outcomes, and this is a crucial factor in environmental adaptation [47]. Adolescents with higher control perceptions show greater agency and more positive evaluations of their abilities and influence on external events [48,49]. This perception is closely linked to self-efficacy, as believing in one’s influence over outcomes enhances confidence in personal capabilities [50]. In a rapidly evolving digital space, adolescents with stronger control perceptions are more resilient to challenges. SGs, with their immersive and risk-free environments, allow adolescents to explore cyberspace, reinforcing their psychological control and self-efficacy.

Feedback mechanisms and progress visualization are central to this process. In SGs, players receive immediate feedback for their actions, which strengthens their sense of control and helps establish a sense of order in digital environments. When unexpected outcomes occur, such as errors, timely prompts and guidance support the acquisition of internet knowledge and skills [51]. Through iterative trial and error, adolescents refine internet skills, ultimately applying them with confidence in real life.

SGs also provide structured environments that support the accumulation of mastery experiences, the most influential source of self-efficacy according to Bandura [43]. Research confirms that robust internet knowledge and experience significantly boost self-efficacy [52]. Therefore, SGs, by providing simulated online environments, allow adolescents to accumulate mastery experiences as they complete tasks, effectively strengthening their sense of online control and self-efficacy. Additionally, SGs set challenging tasks and achievement systems that gradually enhance players’ self-efficacy. In these games, players overcome a series of challenges, complete tasks, and receive rewards, and these positive experiences further enhance their self-efficacy [53]. When adolescents perceive their abilities and accomplishments in the online space, they are more likely to transfer this confidence and motivation to real life, facing challenges in the online environment with greater composure.

In summary, SGs enhance IA through scenario simulations, real-time feedback, and achievement systems. They strengthen adolescents’ sense of control and self-efficacy, equipping them to navigate the digital world with confidence and resilience.

### The Role of SGs in the Sustained Adaptation Stage

In the sustained adaptation stage, SGs consolidate earlier learning and support the transfer of in-game experiences to real-world online contexts, reinforcing adolescents’ self-regulation, control, resilience, and proactive engagement.

SGs use scenario-based instructional design to promote learning transfer, defined as the application of acquired knowledge and skills to new situations [54]. Contextualization involves embedding learning content within a scenario that closely mirrors real-world situations or practical problems [1]. Constructivist theory emphasizes that transfer depends on the alignment between learning contexts and real-world application contexts [55,56], a principle operationalized in SGs through realistic online simulations. For instance, cybersecurity games place players in expert roles, requiring them to manage cyberattacks and digital threats, thereby strengthening practical skill acquisition [57]. Activity theory further suggests that knowledge transfer efficiency depends on how well virtual activities align with real-world contexts [58]. Accordingly, the high contextual similarity between SGs and authentic online environments facilitates the efficient application of in-game learning to real-life digital situations.

Beyond supporting transfer, SGs offer scalable and flexible learning opportunities. Unlike traditional educational models, they do not depend on continuous teacher guidance or fixed learning spaces. Learners can engage in game-based learning activities at their own pace and according to their personal schedules, significantly enhancing learning continuity, flexibility, and autonomy [18]. Additionally, SGs can be updated in real time to reflect emerging technologies and online risks, ensuring that adolescents’ knowledge and skills remain aligned with evolving digital environments [22,23]. By transforming from static tools into dynamic platforms, SGs adapt to the ever-changing online landscape, offering a deeper and more practical experience to enhance IA.

In sum, by combining contextualized simulation, flexible access, and continuous updating, SGs support the sustained development of adolescents’ IA and promote durable, transferable learning outcomes.

### The Dynamic Enhancement Role of SGs Across the 3 Stages

SGs enhance adolescents’ IA not only through the distinct functions of preparation, adaptation, and sustained adaptation, but also through their cyclical and progressive integration. Together, these stages form a dynamic learning mechanism that supports continuous reflection, application, and long-term adaptability.

Self-regulated learning (SRL) theory helps explain this process. Zimmerman [59] conceptualizes SRL as a cycle of planning, monitoring, and reflection that enables learners to regulate their behavior through goal setting, process evaluation, and experience review [53]. These processes align closely with the 3 stages of IA. In the preparation stage, SGs stimulate motivation and initiate the planning phase by encouraging adolescents to set learning goals, such as improving information search or cybersecurity awareness. In the adaptation stage, games support monitoring and strategy adjustment through immediate feedback and adaptive task design, requiring learners to apply prior knowledge in increasingly complex situations [59]. This iterative practice enhances focus, strengthens psychological control, and promotes self-efficacy. In the sustained adaptation stage, SGs consolidate learning outcomes through task progression and knowledge transfer. Reflection becomes especially prominent, as summary modules and performance tracking support evaluation of learning strategies and outcomes [60].

Across these stages, SGs create a spiral learning process. Goal setting in preparation lays the foundation for practice, experiences in adaptation deepen understanding, and reflection in sustained adaptation initiates the next learning cycle [59]. By integrating SRL processes with progressive game design, SGs not only enhance adolescents’ current IA but also cultivate long-term SRL abilities. These skills enable adolescents to continually update and apply knowledge in an evolving digital environment, supporting sustained adaptability.

## From Theory to Practice: Guiding Design Principles

Although SGs offer potential benefits, evidence shows their effectiveness is not guaranteed. Some studies report limited behavioral change or real-world transfer, including health behavior [61], safety knowledge [62], and cognitive or metacognitive skills [63]. In educational settings, games can boost engagement without significant learning gains [27,64], or produce variable effects depending on design [65]. These findings highlight that outcomes depend more on design quality than on the game format itself [66].

The same applies to IA. Games promote adaptive development only when their goals, mechanics, and feedback support the cognitive, emotional, and behavioral processes essential for navigating digital environments. Translating theoretical mechanisms into practical design is therefore crucial. The following section presents key design principles for developing SGs that enhance adolescents’ IA.

### Principle of Clear Goal Definition

Clear goal definition is the foundational principle of IA-oriented SG design, as without explicit objectives, embedded mechanisms cannot reliably translate into meaningful learning outcomes. Within the SRL theory, the clear and specific goals guide learners’ reflection, evaluation, and behavioral regulation throughout the learning process [67]. Constructivist learning theory similarly emphasizes defining the instructional theme for overall design and each unit [67]. However, some designers adopt existing game types without aligning them to specific educational goals, risking misalignment [18,68].

In the context of IA, goal clarity is particularly critical. IA comprises 8 core dimensions [1]: attitude, online knowledge and skills, sense of control, self-efficacy, adaptability, self-control, psychological resilience, and proactivity. These dimensions differ in importance across developmental stages: the preparation stage emphasizes attitudes and foundational skills, whereas the adaptation stage prioritizes sense of control and self-efficacy [1]. Accordingly, game objectives should be sequenced to reflect these developmental priorities.

Additionally, objectives must match adolescents’ cognitive development. Overly complex or insufficiently scaffolded goals can increase cognitive load and hinder schema formation [69,70]. Finally, objectives should also be measurable to support progress monitoring. Standardized tests, surveys, and in-game analytics allow developers and educators to track learning and refine design [19]. Measurable goals also reinforce the SRL cycle by enabling players to compare current performance with desired outcomes and adjust strategies accordingly.

In sum, clear goal definition is not merely an administrative step; it operationalizes the psychological mechanisms underlying IA, transforming abstract adaptability constructs into actionable design elements that provide structural guidance for all subsequent design principles.

### Principle of Interaction Diversity

Following clear goal definition, interaction diversity is the second major principle in IA-oriented SG design. While it supports all 3 stages of IA, its psychological functions align most closely with the preparation stage, where adolescents develop foundational knowledge, constructive attitudes, and early exploratory confidence [1]. Interaction diversity refers to offering multiple meaningful ways to engage with digital content, encouraging learners to interpret and respond to online situations from different perspectives [21,71]. It functions not merely as an interface feature but as a mechanism for deepening cognitive processing, activating schemas, and strengthening early adaptive dispositions.

Varied interactions encourage adolescents to move beyond passive exposure and instead engage in increasingly elaborated cycles of exploration, interpretation, and decision-making [21]. Narrative-driven interactions, for instance, allow learners to engage with unfolding digital events in ways that highlight causality and perspective-taking [20]. Such narrative encounters support the activation and restructuring of social-cognitive schemas, enabling adolescents to develop more nuanced expectations regarding online behavior, interpersonal dynamics, and risk cues [22-24]. Procedural interactions, such as simulated online operations or information-handling tasks, enable the formation of procedural knowledge through repeated practice [23-25], while reinforcing early perceptions of competence and control. Socially oriented interactions situate learners within conversational or collaborative exchanges, modeling constructive digital communication and promoting empathy, negotiation, and responsible participation [45]. Reflective interactions, including prompts and consequence-based feedback, encourage learners to examine action outcomes, strengthening metacognitive monitoring and early risk evaluation [21,63]. Such reflective engagements are especially valuable in the preparation stage, where adolescents are forming their earliest internal standards for online judgment and behavior.

Taken together, interaction diversity enables adolescents to engage with digital scenarios through narrative, procedural, social, and reflective pathways, supporting early IA while laying the cognitive and affective foundation for later adaptation and sustained functioning.

### Principle of Contextual Authenticity

Building on interaction diversity, contextual authenticity is a key principle for SGs enhancing adolescents’ IA. Learning in environments resembling real digital contexts helps learners perceive the relevance of skills and decisions [72]. In such authentic scenarios, adolescents engage in experiential learning: they encounter realistic problems, make decisions, observe consequences, and reflect on outcomes [73,74]. This cycle of action, feedback, and reflection fosters mastery experiences, a primary source of self-efficacy [43,72,73], which in turn supports proactive coping and adaptive decision-making in real online environments [75].

However, far transfer from low-risk game settings to high-stakes real-world situations is often limited [63,65]. Realistic scenarios alone do not guarantee effective application of game-learned strategies. To bridge this gap, SGs should include structured scaffolding that supports cognitive and metacognitive transfer [76]. Increasing in-game task complexity and emotional demands allows learners to practice under conditions closer to real digital pressures [77]. This exposure strengthens stress tolerance and decision stability, allowing adolescents to form resilient strategy–response mappings that can generalize beyond the game. Simultaneously, the metacognitive prompts and reflective exercises encourage learners to articulate their reasoning, examine potential biases, and connect in-game experiences with real life [63]. This process transforms gameplay experiences into conceptual knowledge, an essential condition for far transfer, which requires that learners not merely remember actions but understand underlying mechanisms [60]. Embedding negative feedback or conflict scenarios trains emotion regulation while maintaining effective decision-making [78]. Moreover, exposure to multiple roles and scenarios across digital contexts reinforces strategy application and consolidation, increasing the likelihood of successful transfer [45,66].

In this way, contextual authenticity, when supported by structured scaffolding, functions not merely as a realistic setting but as an integrated mechanism that deepens comprehension, strengthens self-efficacy, and facilitates the flexible application of adaptive strategies.

### Principle of Immediate, Scaffolding, and Explanatory Feedback

In addition to the diversity and authenticity of interaction processes, the results of each interaction are equally important for improving adolescents’ learning experience and motivation in SGs [58]. In SGs focused on IA, feedback should be immediate, explanatory, and scaffolded according to developmental stages, directly fostering adaptive self-efficacy, a strong sense of control, and metacognitive regulation [43,79]. Effective feedback establishes a transparent loop between digital behaviors and their consequences, allowing adolescents to rapidly form accurate mental models of online causal relationships [80].

To cultivate adaptability, feedback must go beyond correctness judgments and act as a dynamic scaffold. During the preparation stage, feedback should be highly supportive and formative, linking actions to positive outcomes to reinforce initial schemata and build foundational confidence [81,82]. For example, successfully verifying a website might trigger feedback that affirms the behavior and explains how it enhances digital safety [83]. As learners progress into adaptation and sustained adaptation stages, feedback should increasingly include corrective and explanatory components following not optimal choices. By simulating plausible negative outcomes and providing guided analysis, these interventions transform errors into low-stakes mastery experiences, a process that is critical for developing resilience and flexible problem-solving skills [84]. From the perspective of self-efficacy theory, these scaffolded feedback cycles are the engine for generating mastery experiences, the source of confidence in managing digital challenges [57,82]. Crucially, by explaining the “why” behind both successes and failures, feedback fosters metacognitive awareness, enabling adolescents to understand not only what to do but how to think about digital situations [63]. This lays the foundation for self-regulation and the strategic transfer of skills to new, real-world online contexts [74].

In sum, this principle redefines feedback from a mere informational tool to a key driver of adaptive psychological development, with in-game interaction contributing to both skill acquisition and the cultivation of beliefs and self-regulatory capacities that sustain long-term IA.

### Principle of Dynamically Adaptive Learning Environment

The principles of clear goals, contextual authenticity, interaction diversity, and immediate feedback provide the framework for game-based learning [85]. To extend this foundation into sustainable adaptability, IA-oriented games should function as dynamically adaptive learning environments, operating at both content and individualized learner levels.

At the content level, dynamic adaptability ensures that the game continually integrates new digital challenges reflecting emerging norms, technologies, and risks [86,87]. Rather than presenting fixed modules, the game introduces novel scenarios over time, supporting repeated experiential learning and adaptive transfer [60]. Updates act as pedagogical extensions of emerging digital realities. For example, modules may present artificial intelligence (AI)–related challenges, such as identifying deepfake content, evaluating algorithmically curated information, or managing interactions with AI-driven platforms, helping adolescents respond to evolving online risks [87]. As players confront these tasks, they are encouraged to revisit earlier strategies, refine their understanding, and rebuild adaptive approaches. This iterative process strengthens metacognitive awareness, self-regulation, and resilience [88,89]. Embedded assessments can further personalize progression by adjusting difficulty or focus based on performance, cognitive style, or competencies. For example, a player who excels in online communication but lacks cybersecurity skills may receive additional tasks focused on privacy management or digital ethics [90].

At the individual level, dynamic adaptability is supported through an intuitive and well-organized interface that matches learners’ cognitive habits and operational routines [70,91]. Such design reduces extraneous cognitive load and enables learners to concentrate on meaningful problem-solving rather than procedural navigation. By lowering technical barriers and offering clear guidance, the interface functions as a scaffold that facilitates efficient information processing, responsible decision-making, and reflective engagement [92,93]. When combined with dynamically evolving content, this learner-centered support helps players apply adaptive strategies in real time and internalize the self-regulatory and metacognitive skills required for navigating complex digital environments.

In sum, the principle highlights how SGs can integrate evolving content with accessible, learner-centered design. This dual adaptability nurtures reflective, self-regulated, and transferable skills, equipping adolescents with the cognitive and behavioral capacities needed for sustainable IA.

### Principle of Safety-by-Design for Digital Well-Being

While the preceding principles focus on fostering motivation, cognitive engagement, and effective skill acquisition, Safety-by-Design ensures that the learning environment itself does not inadvertently reproduce the addictive or compulsive engagement patterns associated with negative forms of internet use [94]. Adolescents are particularly sensitive to reward-seeking cues, variable reinforcement schedules, and emotionally arousing feedback structures, all of which can amplify screen fixation or habitual checking behaviors [95]. To counter these risks, safety-by-design establishes boundaries that promote regulated, purposeful engagement rather than hedonic immersion. For instance, gameplay cycles are intentionally time-bounded and punctuated by reflective pauses that encourage players to monitor their internal states, evaluate the usefulness of the strategies they are using, and recognize the distinction between intentional learning and habitual digital consumption [96]. Moreover, the reinforcement mechanisms embedded in the game prioritize informational over affective feedback: success is communicated through clarity of consequences, improvement indicators, or strategy-relevant insights rather than through escalating stimuli such as points, streaks, or attention-grabbing animations [97]. By avoiding variable structures and other persuasive design elements known to increase compulsive use [98], the game models a healthier digital ecology that aligns with the broader objective of cultivating adaptive self-regulation.

Additionally, Safety-by-Design further supports IA by fostering metacognitive awareness of one’s digital habits [96]. While the structural boundaries of the game help reduce the likelihood of compulsive use, the next step is enabling adolescents to understand and actively manage their own digital habits. Break reminders, self-assessment checkpoints, and reflective prompts, which have been shown to foster self-regulation and transfer in digital learning environments [63,96]. These opportunities for reflection help adolescents identify moments when online interactions may compromise their well-being and consider how the strategies practiced in the game could inform their responses in everyday digital contexts.

Together, the structural safeguards and metacognitive supports enable Safety-by-Design to move beyond simply preventing harmful engagement, actively fostering adolescents’ self-regulation and reflective capacities for adaptive, intentional, and sustainable digital behavior.

## Conclusions and Future Research

This review examined how SGs enhance adolescents’ IA through the 3 stages of preparation, adaptation, and sustained adaptation. By leveraging contextualized design, real-time feedback, and dynamic tasks, these games support experiential learning, psychological control, skill acquisition, and resilience to online risks. Based on these insights, we proposed design principles to guide the development of IA-oriented SGs.

However, this work remains theoretical and requires empirical validation. Future studies should develop and evaluate SGs targeting age-specific adaptability traits across diverse online environments, considering both user experience and educational effectiveness. Additionally, future research could investigate how IA manifests across culturally and digitally diverse contexts. Drawing on ecological and social-ecological frameworks [99], researchers can examine how nested environmental systems, including family, school, peer networks, and broader community and sociocultural contexts, shape both the types of online challenges adolescents encounter and the strategies they use to adapt. These studies would clarify how IA is shaped by different environments and inform culturally sensitive interventions and SG design.

With rapid advances in AI, adolescents face increasing demands, such as evaluating information authenticity and responding to novel digital risks. Future game designs can integrate AI-driven situational simulations and real-time adaptive feedback to personalize learning, simulate AI-generated risks, and help adolescents practice verification, problem-solving, and adaptive strategies [86,87,100].

In conclusion, SGs, as an innovative educational tool, hold great potential for improving adolescents’ IA. Future research should further combine technological innovations with empirical studies to explore their application across varied educational settings. At the same time, effective implementation requires collaboration among policymakers, schools, and parents to build a supportive internet ecosystem. With ongoing efforts, SGs can become an essential tool in adolescent internet education, providing sustained support for their learning and development.

## Abbreviations

AI: artificial intelligence
IA: internet adaptability
SG: serious game
SRL: self-regulated learning

## References

[1] Wang W, Dong R, Niu G, Zhou Z (2021). Network adaptation: concepts and models. J Nanchang Univ (Humanities Soc Sci Ed).

[2] Uchino BN (2009). Understanding the links between social support and physical health: a life-span perspective with emphasis on the separability of perceived and received support. Perspect Psychol Sci.

[3] Tinmaz H, Lee YT, Fanea-Ivanovici M, Baber H (2022). A systematic review on digital literacy. Smart Learn Environ.

[4] Yeşilyurt E, Vezne R (2023). Digital literacy, technological literacy, and internet literacy as predictors of attitude toward applying computer-supported education. Educ Inf Technol (Dordr).

[5] Carretero Gomez S, Vuorikari R, Punie Y (2017). DigComp 2.1: the digital competence framework for citizens with eight proficiency levels and examples of use.

[6] Pan Q, Lan M, Tan CY, Tao S, Liang Q, Law N (2024). Protective factors contributing to adolescents’ multifaceted digital resilience for their wellbeing: a socio-ecological perspective. Comput Human Behav.

[7] Arkan Z, Bal M (2025). The relationship between school-age students’ literacy skills and digital well-being: a systematic review. BMC Psychol.

[8] Wang W, Ma S, Han X, Zhao X (2023). The impact of internet adaptability on internet addiction: the serial mediation effect of meaning in life and anxiety. Front Psychiatry.

[9] Chen X, Wang L, Wang Z (2009). Shyness-sensitivity and social, school, and psychological adjustment in rural migrant and urban children in China. Child Dev.

[10] Jin CC, Wang BC, Ji AT (2019). The relationship between the dark triad and internet adaptation among adolescents in China: internet use preference as a mediator. Front Psychol.

[11] Dong WH, Zhang J, Meng SJ, Jia M, Wang WJ (2025). The topological structure of adolescents’ internet adaptation: a longitudinal tracking study. Acta Psychologica Sinica.

[12] Livingstone S, Helsper EJ (2010). Balancing opportunities and risks in teenagers’ use of the internet: the role of online skills and internet self-efficacy. New Media & Society.

[13] Abt CC (1970). Serious Games.

[14] Franco Vega I, Eleftheriou A, Graham C (2022). Using video games to improve the sexual health of young people aged 15 to 25 years: rapid review. JMIR Serious Games.

[15] Zyda M (2005). From visual simulation to virtual reality to games. Computer (Long Beach Calif).

[16] Lameras P, Arnab S, Dunwell I, Stewart C, Clarke S, Petridis P (2017). Essential features of serious games design in higher education: linking learning attributes to game mechanics. Brit J Educational Tech.

[17] Li F, Sun Y (2019). Functional games: definition, value exploration, and development suggestions. Educ Media Res.

[18] Tay J, Goh YM, Safiena S, Bound H (2022). Designing digital game-based learning for professional upskilling: a systematic literature review. Comput Educ.

[19] Caserman P, Hoffmann K, Müller P (2020). Quality criteria for serious games: serious part, game part, and balance. JMIR Serious Games.

[20] Naul E, Liu M (2020). Why story matters: a review of narrative in serious games. J Educ Comput Res.

[21] Fonseca X, Slingerland G, Lukosch S, Brazier F (2021). Designing for meaningful social interaction in digital serious games. Entertain Comput.

[22] Calvo-Morata A, Alonso-Fernández C, Freire M, Martínez-Ortiz I, Fernández-Manjón B (2020). Serious games to prevent and detect bullying and cyberbullying: a systematic serious games and literature review. Comput Educ.

[23] Hart S, Margheri A, Paci F, Sassone V (2020). Riskio: a serious game for cyber security awareness and education. Computers & Security.

[24] Ahmead M, El Sharif N, Abuiram I (2024). Risky online behaviors and cybercrime awareness among undergraduate students at Al Quds University: a cross sectional study. Crime Sci.

[25] Booc NBB, Budiongan K, Carballo R (2024). Cybersecurity awareness, and cybersecurity behavior of high school students in Davao city: a mediation role of perceived behavioral control. Eur J Appl Sci Eng Technol.

[26] Wang W, Li J, Liu S, Ye J, Ma S (2025). Development and validation of an adolescent internet adaptability educational game based on situated learning theory. Educ Inf Technol.

[27] Riopel M, Nenciovici L, Potvin P (2019). Impact of serious games on science learning achievement compared with more conventional instruction: an overview and a meta-analysis. Studies in Science Education.

[28] Behl A, Jayawardena N, Pereira V, Islam N, Giudice MD, Choudrie J (2022). Gamification and e-learning for young learners: a systematic literature review, bibliometric analysis, and future research agenda. Technol Forecast Soc Change.

[29] Ullah H, Afzal S, Khan IU (2022). Perceptual quality assessment of panoramic stitched contents for immersive applications: a prospective survey. VRIH.

[30] Yu Z (2023). Learning outcomes, motivation, and satisfaction in gamified english vocabulary learning. Sage Open.

[31] Almeida F, Buzady Z (2022). Development of soft skills competencies through the use of FLIGBY. Technol Pedagog Educ.

[32] Ștefan IA, Hauge JB, Hasse F, Ștefan A (2019). Using serious games and simulations for teaching co-operative decision-making. Procedia Comput Sci.

[33] Bakhanova E, Garcia JA, Raffe WL, Voinov A (2020). Targeting social learning and engagement: what serious games and gamification can offer to participatory modeling. Environ Model Softw.

[34] Tsai CC, Lin SSJ, Tsai MJ (2001). Developing an Internet Attitude Scale for high school students. Comput Educ.

[35] Tu W, Wang M, Li Y, Nie Y (2022). The relationship between college students’ network attitudes and internet addiction: a chain mediation effect. Chin J Health Psychol.

[36] Brandtzæg PB (2010). Towards a unified media-user typology (MUT): a meta-analysis and review of the research literature on media-user typologies. Comput Human Behav.

[37] Deng Y, Zhang Y, Zhao R (2025). Investigating learning behaviors in desktop-based simulated and VR headset-based immersive 3D learning environments: a cross-media comparative study. Educ Inf Technol.

[38] Makransky G, Petersen GB (2021). The cognitive affective model of immersive learning (CAMIL): a theoretical research-based model of learning in immersive virtual reality. Educ Psychol Rev.

[39] Anderson RC, Alvermann DE, Unrau NJ, Sailors M, Ruddell RB (2018). Theoretical Models and Processes of Literacy.

[40] Jung E, Lim R, Kim D (2022). A schema-based instructional design model for self-paced learning environments. Education Sciences.

[41] Ye L, Zhou X, Yang S, Hang Y (2023). Serious game design and learning effect verification supporting traditional pattern learning. Interactive Learning Environments.

[42] Petersen GB, Petkakis G, Makransky G (2022). A study of how immersion and interactivity drive VR learning. Comput Educ.

[43] Bandura A (1997). Selfefficacy: The Exercise of Control.

[44] Ferreira PC, Simão AMV, Paiva A, Martinho C, Prada R, Rocha J (2022). Serious game-based psychosocial intervention to foster prosociality in cyberbullying bystanders. Psychosoc Interv.

[45] Bachen CM, Hernández-Ramos P, Raphael C, Waldron A (2016). How do presence, flow, and character identification affect players’ empathy and interest in learning from a serious computer game?. Comput Human Behav.

[46] Gentile DA, Anderson CA, Yukawa S (2009). The effects of prosocial video games on prosocial behaviors: international evidence from correlational, longitudinal, and experimental studies. Pers Soc Psychol Bull.

[47] Skinner EA (1996). Perceived Control, Motivation, & Coping.

[48] Ryan RM, Deci EL (2000). Self-determination theory and the facilitation of intrinsic motivation, social development, and well-being. Am Psychol.

[49] Frazier P, Keenan N, Anders S, Perera S, Shallcross S, Hintz S (2011). Perceived past, present, and future control and adjustment to stressful life events. J Pers Soc Psychol.

[50] Li Y (2016). The relationship between college students’ locus of control, academic selfefficacy, and learning autonomy [master’s thesis]. https://kns.cnki.net/kcms2/article/abstract?v=oGHOruzuSDOsSFlE8FkgRSdigHGEsA5dZKh67brXPh6mYbPHk1GR79vVnorTBc9zyj1nCGMx3mHOmYecrmagOb_ou2OnGoHim5y35Ml1g7L3Qp2oyNdNoF10ST0cCdpl72784ipbgD58fXALTgJxNhUdcSMyuXl_6uzaQC9tsr87Q9mYyLmgAA==&uniplatform=NZKPT&language=CHS.

[51] Hamari J, Koivisto J, Sarsa H Does gamification work? -- A literature review of empirical studies on gamification.

[52] Anderson AA, Brossard D, Scheufele DA, Xenos MA, Ladwig P (2014). The “Nasty Effect:” online incivility and risk perceptions of emerging technologies. J Comput-Mediat Comm.

[53] Arnab S, Lim T, Carvalho MB (2015). Mapping learning and game mechanics for serious games analysis. Brit J Educational Tech.

[54] Prenzel M, Mandl H, Frensch PA, Funke J (1993). Complex Problem Solving: The European Perspective.

[55] Brown JS, Collins A, Duguid P (1989). Situated cognition and the culture of learning. Educ Res.

[56] Barnett SM, Ceci SJ (2002). When and where do we apply what we learn? A taxonomy for far transfer. Psychol Bull.

[57] Roque C, Moodley G, Mandal S (2024). Cybersafe: gamifying cybersecurity training with a training app. iccws.

[58] Hamari J, Shernoff DJ, Rowe E, Coller B, Asbell-Clarke J, Edwards T (2016). Challenging games help students learn: an empirical study on engagement, flow and immersion in game-based learning. Comput Human Behav.

[59] Zimmerman BJ (2002). Becoming a self-regulated learner: an overview. Theory Pract.

[60] Sailer M, Homner L (2020). The gamification of learning: a meta-analysis. Educ Psychol Rev.

[61] DeSmet A, Van Ryckeghem D, Compernolle S (2014). A meta-analysis of serious digital games for healthy lifestyle promotion. Prev Med.

[62] Dankbaar MEW, Richters O, Kalkman CJ (2017). Comparative effectiveness of a serious game and an e-module to support patient safety knowledge and awareness. BMC Med Educ.

[63] Zumbach J, Rammerstorfer L, Deibl I (2020). Cognitive and metacognitive support in learning with a serious game about demographic change. Comput Human Behav.

[64] Tsai YL, Tsai CC (2020). A meta‐analysis of research on digital game‐based science learning. Computer Assisted Learning.

[65] Gui Y, Cai Z, Yang Y, Kong L, Fan X, Tai RH (2023). Effectiveness of digital educational game and game design in STEM learning: a meta-analytic review. IJ STEM Ed.

[66] Clark DB, Tanner-Smith EE, Killingsworth SS (2016). Digital games, design, and learning: a systematic review and meta-analysis. Rev Educ Res.

[67] Jonassen DH, Reigeluth CM (1999). Instructional-Design Theories and Models: A New Paradigm of Instructional Theory.

[68] Connolly TM, Boyle EA, MacArthur E, Hainey T, Boyle JM (2012). A systematic literature review of empirical evidence on computer games and serious games. Comput Educ.

[69] Plass JL, Homer BD, Kinzer CK (2015). Foundations of game-based learning. Educ Psychol.

[70] Sweller J, Ayres P, Kalyuga S (2011). Cognitive Load Theory.

[71] Chen CW (2025). Low-tech serious games in higher education: bridging the digital divide and enhancing student thinking and performance. Humanit Soc Sci Commun.

[72] Merchant Z, Goetz ET, Cifuentes L, Keeney-Kennicutt W, Davis TJ (2014). Effectiveness of virtual reality-based instruction on students’ learning outcomes in K-12 and higher education: a meta-analysis. Comput Educ.

[73] Kolb DA (2015). Experiential Learning: Experience as the Source of Learning and Development.

[74] Ferchaud A, Beth Oliver M (2019). It’s my choice: the effects of moral decision-making on narrative game engagement. J Gaming Virtual Worlds.

[75] Lee AY, Hancock JT (2023). Developing digital resilience: an educational intervention improves elementary students’ response to digital challenges. Computers and Education Open.

[76] Nietfeld JL, Anderson CA, Yukawa S (2020). Predicting transfer from a game-based learning environment. Comput Educ.

[77] Richter K, Kickmeier-Rust M (2025). The role of gamification in learning transfer: does early skill learning predict performance in complex tasks?. ECGBL.

[78] López-Serrano A, McGowan N, Moreno-Ger P, Burgos D (2025). Teaching soft skills in higher education through serious games: validation of the compete! gamification. Smart Learn Environ.

[79] van der Kleij FM, Eggen T, Timmers CF, Veldkamp BP (2012). Effects of feedback in a computer-based assessment for learning. Comput Educ.

[80] Maxim RI, Arnedo-Moreno J (2025). Identifying key principles and commonalities in digital serious game design frameworks: scoping review. JMIR Serious Games.

[81] Andersson C, Granberg C, Palmberg B, Palm T (2025). Basic psychological needs satisfaction as a mediator of the effects of a formative assessment practice on behavioral engagement and autonomous motivation. Front Educ.

[82] Guay F (2022). Applying self-determination theory to education: regulations types, psychological needs, and autonomy supporting behaviors. Can J Sch Psychol.

[83] Ravyse WS, Seugnet Blignaut A, Leendertz V, Woolner A (2017). Success factors for serious games to enhance learning: a systematic review. Virtual Real.

[84] Yasin A, Liu L, Li T, Fatima R, Jianmin W (2019). Improving software security awareness using a serious game. IET Software.

[85] Dabbous M, Kawtharani A, Fahs I (2022). The role of game-based learning in experiential education: tool validation, motivation assessment, and outcomes evaluation among a sample of pharmacy students. Education Sciences.

[86] Pistono A de A, dos Santos AMP, Baptista RJV, Mamede HS (2024). Framework for adaptive serious games. Comp Applic In Engineering.

[87] Tolks D, Schmidt JJ, Kuhn S (2024). The role of AI in serious games and gamification for health: scoping review. JMIR Serious Games.

[88] Checa-Romero M, Gimenez-Lozano JM (2025). Video games and metacognition in the classroom for the development of 21st century skills: a systematic review. Front Educ.

[89] Zheng R, New J (2017). Handbook of Research on Serious Games for Educational Environments.

[90] Vanbecelaere S, Demedts F, Reynvoet B, Depaepe F (2023). Toward a framework for analyzing adaptive digital games’ research effectiveness. IJSG.

[91] Carmichael L, Poirier SM, Coursaris CK, Léger PM, Sénécal S (2022). Users’ information disclosure behaviors during interactions with chatbots: the effect of information disclosure nudges. Appl Sci (Basel).

[92] Carrión-Toro M, Morales-Martínez D, Santórum M (2025). Rethinking usability in serious games: designing an instrument that evaluates what really matters in learning contexts. Appl Sci (Basel).

[93] Sweller J, van Merriënboer JJG, Paas F (2019). Cognitive architecture and instructional design: 20 years later. Educ Psychol Rev.

[94] Spahl W, Motta V, Woodcock K, Rubeis G (2024). Gamified digital mental health interventions for young people: scoping review of ethical aspects during development and implementation. JMIR Serious Games.

[95] Batten SR, Bang D, Kopell BH (2024). Dopamine and serotonin in human substantia nigra track social context and value signals during economic exchange. Nat Hum Behav.

[96] Shaheen A, Ali S, Fotaris P (2023). Assessing the efficacy of reflective game design: a design-based study in digital game-based learning. Education Sciences.

[97] Karhulahti VM (2024). Vitality structures in “addictive” game design. Open Res Eur.

[98] Saini N, Hodgins DC (2023). Investigating gaming structural features associated with gaming disorder and proposing a revised taxonomical model: a scoping review. J Behav Addict.

[99] Bronfenbrenner U (2005). Making Human Beings Human: Bioecological Perspectives on Human Development.

[100] Kowal M, Conroy E, Ramsbottom N, Smithies T, Toth A, Campbell M (2021). Gaming your mental health: a narrative review on mitigating symptoms of depression and anxiety using commercial video games. JMIR Serious Games.

