# Correction: The Mechanism and Design Principles of Serious Games in Enhancing Adolescents’ Internet Adaptability

**DOI:** 10.2196/100612

**Published:** 2026-07-31

**Authors:** Shijie Gao, Min Jia, Weijun Wang, Jianping Hu, Shihao Ma

**Affiliations:** 1 School of Psychology, Central China Normal University, No.152 Luoyu Road, Hongshan District, Wuhan, Hubei, 430079, China, 86 18560177867; 2 Key Laboratory of Adolescent Cyberpsychology and Behavior, Ministry of Education, Wuhan, Hubei, China

In “The Mechanism and Design Principles of Serious Games in Enhancing Adolescents’ Internet Adaptability” [1], the authors noted one error. Equal contribution with Min Jia was incorrectly attributed to Shihao Ma instead of Shijie Gao.

The author list previously appeared as follows:

*Shijie Gao* ^*1*^ *, BS; Min Jia* ^1*^ *, MA; Weijun Wang* ^*2*^ *, PhD; Jianping Hu* ^*1*^ *, MA; Shihao Ma* ^1*^ *, PhD*

It has been corrected to:

*Shijie Gao* ^1*^ *, BS; Min Jia* ^1*^ *, MA; Weijun Wang* ^*2*^ *, PhD; Jianping Hu* ^*1*^ *, MA; Shihao Ma* ^*1*^ *, PhD*

The correction will appear in the online version of the paper on the JMIR Publications website, together with the publication of this correction notice. Because this was made after submission to PubMed, PubMed Central, and other full-text repositories, the corrected article has also been resubmitted to those repositories.
